# Christmas Appeal Supplement

**Published:** 1899-12-16

**Authors:** 


### CHRISTMAS APPEAL SUPPLEMENT.

### Our Queen.

(WITH TWO PORTRAITS AND AUTOGRAPHS.)

### THE QUEEN-MOTHER.

A wise mother, a loving mother, a mother not without some traits of sternness—such is our Queen, the mother of a loving people, the people of England and of the Empire. None other has ever united in one personality so glorious an example of motherhood and sovereign greatness. The union has produced the best sovereign who has ever graced the British throne, and it has secured her the warmest love of the whole people of a mighty Empire. Gradually, but without intermission, has the love of her people for their Queen increased during the longest reign in English history. Respect for the wise sovereign succeeded sympathetic interest in the Girl Queen, and has culminated in enthusiastic love and devotion for Victoria, the venerable Queen-Mother.

It is in her character of mother of her people that the foundation of the greatness of Victoria as a sovereign lies. From the moment that she left the happy seclusion of her girlish life, and entered upon her duties as our Queen, the one aim of Victoria has been the well-being and happiness of her subjects. As a sovereign she will leave behind her an admirable example of diligent attention to duty, of wise conduct, and of subordination of private inclinations and opinions to the public good. As a mother she will serve as a model of virtue, tenderness, and sympathy. It is difficult to over-estimate the influence which the Queen-Mother has exerted over us as a nation. Who dare say that the standard of virtue held up by a sovereign has not served to influence public opinion in the direction of all that is good and right? and public opinion is the lever whereby reform is gradually effected.

The Queen's sympathy and charity have been amongst the brightest jewels in the queenly diadem. How has the example been followed? In a manner which must gladden the heart of the venerable Queen-Mother, though she may not recognise how great a part she has played in the result. Throughout the length and breadth of her Empire succour for sufferers from every conceivable misfortune abounds. The generosity is boundless; the want has no sooner to be known than the givers step forward with their offerings. And, best of all, the charity and the sympathy are becoming, as time goes on, more closely united in the givers. The Queen and her children are largely responsible for this. Their gifts have never been doles; they are made with sympathetic feeling for the requirements of every case, and the same spirit is apparent in all who come under their influence. Just at this moment the effect of the example set by the Queen-Mother is strikingly illustrated throughout the nation. Not only have vast sums of money flowed from all sides to assist the sufferers from the war, but everywhere kindly thoughts have been at work to provide comforts for our soldiers, such as no central organisation could secure to them. Grateful as, no doubt, our soldiers and sailors will be for all the generosity exercised towards them, it is probable that nothing will give such real pleasure as the Tam o'Shanters, the tobacco, and, above all, the touching little personal present of the Queen-Mother.

Whilst her love for all her people has been most evident throughout her life, in no direction has it been more apparent than towards her Army and Navy. In one of her letters many years ago, after witnessing the departure of a squadron of ships for the Baltic, she wrote: "I am very enthusiastic about my dear Army and Navy, and wish I had two sons in both now. I know I shall suffer much when I hear of losses among them." And, again, after laying the foundation-stone of Netley Hospital, one of her letters contains these words: "Loving my brave Army as I do, and having seen so many of my poor sick and wounded soldiers, I shall watch over this work with maternal anxiety." Forty years and more have passed since these words were written. How true to them, how true to her maternal feelings, she has shown herself her loving subjects know full well. The picture of the aged Queen uttering words of sympathy and encouragement to her wounded soldiers from her chair in the corridors of that same Netley Hospital is still fresh in our memory. Fresher still is the impression of her words to the American nurses who are going to South Africa. Very simply, but from the heart of the Queen-Mother came the words: "Thank you for coming to nurse my men." In times of trouble no expressions of concern go to the heart more than those which come from the Queen-Mother. Nor are they ever wanting. Her hearty approval when acts of valour reach her ears is the best encouragement to her soldiers. They know that whilst her motherly heart bleeds at the sad death-roll, death in the cause of Queen and country is a lot no true soldier of the Queen will shrink from. None will hasten sooner to comfort those left behind than the Queen, whose heart is chastened by the losses of those nearest and dearest to herself.

In the homes of England the name of the Queen-Mother is venerated. She has exalted the sanctity of family life, and her children live to call her blessed. Gratitude, respect, and affection are hers throughout the Empire as in her family circle. There she has never ceased to exercise her influence alike to the benefit of her descendants and of her own people. The attitude of the German Emperor towards his venerable grand-mother is a striking instance of this, a touching trait in his character, and a genuine tribute to our Queen. At all times may her children and her children's children ever rally round her, to give some consolation to the tender heart which, from the experience of its own sorrows, feels more keenly the affliction of others. Every member of her family and all her people earnestly desire that only happy years may attend the Queen-Mother.

### The Queen-Empress.

We have ventured to summarise a speech made by Lord Rosebery at the Queen's birthday celebration, May 24th, 1899, because it contained an eloquent, forcible, and accurate tribute to the majesty and motherliness of the Queen.

"When the Queen came to the throne of this Empire in 1837, greatly less—incomparably less—though it was in extent and in population than it is now, yet in mounting that historical and immemorial throne she at once obtained, in her eighteenth year, all the honour, all the power, all the splendour that the world could give. . . . Yet our Queen has not been merely Queen of the United Kingdom of Great Britain and Ireland, Empress of India, and Sovereign head of the Colonies. She has been much more than this. By giving to the world the example, in the highest place, of the purest and loftiest life she has exercised a sovereignty far beyond the limit of her own dominions, which can be bounded only by those who appreciate purity and faith in high places. She has fixed—and fixed, so far as we can see, for ever—this ancient monarchy and preserved this Empire, which she has also so greatly extended. When, in 1848, all the thrones of Europe were convulsed, and some of the Sovereigns of Europe were only too anxious to cross the British Channel and find themselves in security here, the throne of Great Britain almost alone in the shock stood undisturbed owing to two facts. One was the emancipating policy of the great Sir Robert Peel, and the other was the influence and position of the Queen. And what is she, not merely with regard to the preservation of the monarchy, but with regard to the maintenance of the Empire, outside these islands, of which so little was said and thought at the commencement of her reign? Great as this Empire is, vast as is its wealth, inexhaustible as are its resources, unbounded—and that is, perhaps, most important of all—as is its influence, what is the bond that unites it to the one hundred, the two hundred, the innumerable millions of India, the English, the Anglo-Saxon, population, scattered all over the world—some with one particular form of government and some with another, some with one form of Constitution and some with another, some with one tariff and some with another—what is the bond that connects all these? What but the Queen? The Queen is the bond and the symbol of the Empire.

"No one who has seen two jubilees of her Majesty can doubt that fact—can doubt that if under other auspices and under worse Sovereigns a different form of government was ever to be established in these islands, not merely would much of our essential freedom be diminished, but our Empire outside these islands would cease to exist. Not merely has the Queen done all here claimed for her, she has done something more. She has strengthened the cause of monarchy all over the world. It is not her own throne that she has established on a base incomparably broader and stronger than that of any other throne in the world, but she has strengthened all righteous thrones, all upright monarchs; and probably in the United States to-day we could find hundreds of thousands—nay, millions—of upright, ardent, and convinced Republicans, opposed to monarchy in every shape and form, who would make one reservation, both mental and open—that of the Sovereign of these islands and the throne she occupies. It is not known by what name our Queen will be known in history—it is too early to anticipate that verdict; but at least it can be said by what name and in what terms she is known to us—as Victoria the venerable and Victoria the well-beloved. She at any rate has earned any term of affection, any term of popularity that her subjects can give her, for she has not merely maintained and raised the monarchy, she has not merely preserved and increased the Empire, she has not merely maintained, but has raised the standard of morals in her country, she has not merely, as far as her Government can, preserved the peace, and increased the peaceful development of the Empire, but she has raised the estimate of human nature itself. We have been privileged to see how a young girl, exposed to all the splendour and temptation of the greatest of thrones, has been able to preserve to her 80th year a reputation not merely unspotted—for that would be little—not merely unclouded—for that would not be much—but one that has, to my belief, exalted human nature itself. This epoch will live in history as one of the brightest which history has recorded. Thrones and Powers, dominions and sceptres crumble and pass away, as history tells us. This pure and powerful reign will not pass away—it will remain embedded in history for long centuries to come, and will have its influence over generations yet unborn and countries that perhaps have yet to spring into existence under the auspices of the Anglo-Saxon race."

### On Wings of Love: An Angelic Visitation.

Written by Nurses.

### The Angel's Mission.

The snow had been falling softly all day, but when the evening came, as if afraid of the darkness, the flakes had fled away, and now one by one the stars began to peep forth. The heavy clouds, which had hung as a pall over the dull earth, had retired to the West, where they remained heaped up in a black mass, showing all the more distinctly against the blue sky which, in departing, they had disclosed to view. Soon the rising moon stole slowly up the sky, tipping the snow-clad boughs with silver sheen as she went, so that all the world, with its sin and its blackness, looked, for once, like a fairy land where all was fair and innocent.

So thought a little child as he walked quietly across the common on his way to his mother's cottage. His feet were very weary, for he had been running errands all day, and his food had been scanty, but his spirit was too full of happiness to care, as he murmured to himself, dragging one little foot after the other, "The Christ-child will come to-morrow! the Christ-child will come to-morrow!"

And then a strange thing happened. Suddenly, just where the largest star was shining steadily down, he saw that he had been mistaken in fancying that all the snowflakes had left, for two much larger than any he had seen before came dropping gently, gently, down to earth. As they came nearer he grew conscious that they shone as no other snowflake had ever shone before, and that on each side a pair of outstretched wings were waving noiselessly through the still air, as the forms came towards him. At last, they were so close that he could almost have touched them; two Angels, their face radiant with the beauty of goodness, yet serious and a trifle sad, as if the sorrow which lay beneath their feet had made their spirits grieve. But their eyes were sweet and loving, and the light which encircled them as they hovered above the boy was like the clear white light of the moon herself. As he gazed, he no longer felt tired and worn out; all the aching had passed away, and his whole soul seemed absorbed in listening to the voices of the Angels as they spoke. He had never heard such a sound before; it reminded him of the big organ in church when the notes were soft and far away.

"And now," said the first Angel, "we must part. My mission is to go into the alleys and streets of the big towns, where vice, and drink, and want have stolen away the love of God from men's hearts, and whisper of that Christmas, 1,900 years ago, when a loving Father sent a little Babe into the world to speak of peace and goodwill. The children delight in my coming, and I am sure of a home in their hearts, but the others—they have no time to listen."

"My mission," said the second Angel, as, with a tremor in her voice, her companion paused, "is a happier one, I think. At least, I love it more. I go in and out the hospitals, wherever suffering, sickness, or pain are present, and help the poor stricken ones to be brave, and patient, and unselfish. And I come across many a humble hero, many an uncomplaining martyr, and I thank God every time I find a soul shining for Him."

And with a quiet bending of their beautiful heads, the Heavenly Visitors flew away. But the child watched them till he could see them no longer, and then, with a hardly perceptible sigh, he turned and walked quietly home. That evening, as his mother worked on after the little ones were in bed, she wondered why her boy's face had such a happy light, or why he whispered, as he kissed her good night, "Perhaps they will come and see you, too, dear, when you go to sleep. They are so sweet, so beautiful."

And the mother thought her son was dreaming already, and patted him softly as she turned away—but, the Angels knew.

### Old Johnny's Love Story.

"The Yule-log sparkled keen with frost,
No wing of wind the region swept,
But over all things brooding slept
The quiet sense of something lost."

The grey blocks of the magnificent building were brightened up and bereft of some of their grimness by a gentle powdering of snow, which glistened in the moonlight. The windows glowed with a warmer radiance as the Angel whispered above this habitation of human suffering her accustomed blessing.

"Peace be to this house, and to all who dwell in it—peace." Then she entered the ward.

It was long and lofty, the walls toned a pleasant, restful green. There were 24 beds, with a window between each. Palms and other evergreen plants grew in pots on the polished window-sills. There were also plants on the bright centre tables, and a few choice flowers for Christmas. A grand piano lent an air of homelike comfort, almost of luxury, to the room. At the far end a cheerful fire was burning brightly, and the yule-log crackled merrily. On couches and invalid chairs a group of convalescent patients had gathered round the fire. It was a busy time for the nurses, some of whom were nursing, others decorating, all doing their utmost to get through the work that lay before them during the next three or four hours. One nurse in the lilac print, white apron, and soft cap of the "Nightingale School" was endeavouring, with the help of two students who had given their services, to make a scroll on Turkey-red with letters of cotton-wool. But at present there was a discussion going on as to what words would be suitable. "A Merry Christmas?" "Well, hardly!" "Welcome?" "Welcome to what? No, not that either."

"I'll tell ye a motto," said old Johnny Grey, a white-haired old man with a rugged, weather-beaten face, who was sitting with his leg on a rest, enjoying a quiet smoke. "Here's a good word for ye, 'East or West, Hame's best!'"

The nurse turned suddenly away so that none but the Angel could see the glistening of the tears which sprang all unexpectedly to her eyes. One of the students began, somewhat inconsequently, to whistle the "Washington Post," and the other said that he had lost his pocket-knife, and began searching for it in unlikely places. But Johnny Grey was started upon a favourite theme, and he was not without listeners. There was the eager, excitable man, with his long, nervous, twitching fingers, and eyes that appeared to be starting from their sockets; and the wasted, phthisical patient, only two-and-twenty, rapidly dying; but daily expecting to recover and go back to work. There was poor wee Jimmy, with chronic asthma, and the gentle little man from Glasgow, always ready to lend a helping hand or a willing ear. There was Eriksen, the great Swedish sailor, with his fine slow eyes and handsome countenance, blessed with a poetic temperament and cursed with chronic rheumatism. There was Geordie from the Tyneside, twisted like a gnarled tree from undue work in childhood. All these sat round and listened as old Johnny spoke.

"Ay, ay," said the old man. "'East or West, Hame's best.' My mother put it up for us every New Year in pink twisties o' paper and green leaves. I mind it was always the same when I was a wee laddie, and syne when I was a grown man. Puir auld mither! she left us lonely when she was ta'en. I got a good place on the railway. It was a wee bit line up north, and I was signalsman. Faith! there's mony a winter nicht I'd 'a gi'en my two lugs for a fire like that. It was a lonesome life after I lost my mither, but I was never wanting a Freend."

The Angel understood of Whom the old man spoke, and so did the Swede, for he tapped Johnny on the shoulder, and quoted a line in broken English from Sankey's hymn—

"What a Friend we have in Jesus."

"And did ye never think o' getting married?" inquired Geordie from Jarrow, after a pause; "there's warse things in the warld than a cannie bit wifie, ye ken."

"Eh, eh," sighed the old man.

"It's a vera seengular theng," said the Glasgow man, in his soft, cooing voice; "but ye'll hardly find a man, or a woman either, that hesna had a love affair o' ain sort or anither."

This was too much for old Johnny, and he renewed his reminiscences.

"There was aince a lass—Faith, faith; but its lang sin' syne. She was straicht and fair as a willow wand, and they called her Jessie, jist Jessie, or whiles Jess—

"She was blithe as she's bonnie,
And modest as ony,
And guileless simpleecity marked her its ain'."

Johnny's head shook a little, and he laid down his pipe.

"But I was never to call her my wife, puir lassie. She lived in a wee bit hoose by the burnside, and I could see her cannie in her gairden fra' my signal-box. Many a Sabbath evening hae we wandered in the glens and spoken o' the day when we should be wed; but the Lord aye kens best."

Here the student, who had by this time found his knife and was listening to Johnny's tale, made a hasty note in his pocket-book and handed it to the nurse, who was rubbing the Swede's shoulder with A B C. She nodded and smiled, and the student went to work again at the scroll.

"It was this way," continued Johnny, "that we never got wed. There was one nicht there was a sair fog. I turned on my reed licht for the north express, and then somehow there came a luggage in the road. I put back the signals, but it was ower late, and there was an accident. I ran doon to do the best I could, but my leg got jammed between twa trucks, so that I couldna move, and the pain was something serious. I was just fainting awa' when my Jessie's face appeared to me like an angel's, and she smiled sae cannie and said, 'there's help coming, Johnny, my man,' and then I lost my senses. Well, I was took doon to the cottage, and Jessie and her mither nursed me better. The railway folks was guid to me on account o' the fog being sae sair that it wasna my blame, so I kept my post. But Jessie had ta'en a chill in the fog, and she got a sair hoast (cough), so that she lay a lang while, and when the spring came she was deed. I never took up wi' ony other woman; we just laid her away in the kirkyard, and ower the grave I set a stane wi' the words, 'East or West, Hame's best'; for weel I kent she was hame, puir lassie, and I'd be meeting her by and by. I kept the place twenty year after that, and then my bad leg took a badness in it that 'ud no mend. I've been in the surgical wards wi' it, but the Professor says it's my constitution, and they fetched me in here. I doubt it'll no be lang till I win hame, and Jessie will be there tae meet me 'by the Fountain.'"

Here Johnny's tale came to an end, for it was bed time; and so the evening wore on to a close. The day nurses turned down the lights and went off. Then the Angel passed softly up and down the ward, giving sleep to those to whom it was sent, patience to the sleepless, and rest and peace to all, that blessed Christmas night.

In the morning the sun shone gaily in upon the evergreens and flowers and Christmas berries, reminding the sufferers that a happier day than usual was in store for them. Over the chimney-piece at the end of the ward hung the scroll—white woolly words on a red ground bordered with laurel leaves—a simple motto, simply executed: "The Lord aye kens best."

### ONE TOO MANY.

It was to the Children's Hospital that the Angel most loved to come. Here she did not come unseen, or pass away unnoticed. In many of the tiny cots ranged round the walls there were eyes that watched her coming, lips that smiled in answer to her smile, small pathetic faces that lighted up as she passed down the long ward.

For little children can see the Angels who are invisible to our eyes, clouded with sin and sorrow and world-weariness.

Everything in the hospital was at its brightest and best on the afternoon of Christmas Eve, and by special permission there were visitors in the wards, when the Angel entered. Fathers and mothers who had come to bring the little patients some small Christmas remembrance; brothers and sisters who looked with half envious eyes at the gaily decorated wards, and listened with wholly envious ears to the prophecies of wonderful presents to come, and of a possible Christmas tree.

"Ain't there a lot of Christmas in a 'orspital; wish I was in 'ere," one small boy said regretfully to his mother. "Such a lot o' flowers and 'olly, and all; and ain't all the kids goin' to 'ave a bully lot o' presents, too; it's a fair nice place this is."

Everyone in the ward was smiling and happy as the Angel went on her way. Peace and goodwill shone from the faces of sisters and nurses, the wardmaids beamed radiantly upon a cheerful world; even the patients who were most ill seemed to have perked up for the occasion, and to be looking their very best.

Did I say everyone? I am wrong. There was one corner of the great medical ward in which there was no brightness—a corner towards which the Angel hastened her steps, because she saw only sorrow there, and she goes most quickly where there is sorrow, that she may bring with her one of her beautiful gifts to make the sorrow less!

A woman sat beside the cot in the corner, a woman in wretchedly poor clothes, holding in her arms a tiny, tiny baby, whilst a child of six clung to her skirts. Her face was worn and thin; she looked hungry and tired, and very, very sad. Her eyes were fixed on the face of the child in the cot: their expression was one of dull misery and of bitterness, rather than grief. The little boy in the cot was scarcely more than a baby, but his face was worn, like the face of an old, old man; the eyes that met the woman's were full of a weary knowledge of the seamy side of life. Such a pathetic scrap of humanity he was, poor baby! Such a forlorn little waif!

The Angel's look was full of pity as she turned from the gay smiling faces at one end of the ward, to this sad little corner at the other. A nurse came softly to the woman's side, and spoke to her kindly.

"Joey seems a wee bit better, Mrs. Martin," she said.

"Do 'e?" The woman's voice was apathetic. "Pore little feller, 'e was pretty nigh starved when 'e was took ill. Me and the kids we ain't doin' very well, not now their dad's at the war; me not bein' on the strength, it make times 'ard."

She looked again at the small pinched face on the pillow. "I don't know whatever I *shall* do when Joey comes 'ome. There ain't enough to go round now, and that's the truth."

She relapsed into dull silence again, and the weary eyes from the cot watched her wistfully.

"Seems as if there was too many of us," the mother went on. "Joey, 'e's all skin and bone, through not never 'avin' enough to eat. And when 'e comes 'ome a-crying for food again, I don't know what I'll do."

"Are times so bad as that?" the nurse asked.

The woman nodded.

"There's too many of us," she reiterated heavily, "and their dad bein' away, and me not on the strength, it makes things 'arder, and the new baby and all. There ain't no 'appy Christmas for me and the kids. There's one less mouth to feed, Joey bein' in the 'orsepital; but whatever 'ull I do when 'e comes 'ome?"

Poor little Joey! Unwanted, uncared for, tired of this great world before he had learnt to know any of its brighter aspects.

His mother rose slowly and wearily, and kissed him perfunctorily ere she departed. The nurse looked after her, a growing wonder passing through her mind over this apparent dulling of all motherly love and tenderness in the struggle of grinding poverty.

The Angel stooped and whispered to the nurse, and a sudden thought flashed into her mind. With quick steps she followed the drooping, forlorn form of the woman down the ward, and outside the door she spoke to her again.

"Mrs. Martin," she said gently, taking a battered little purse from her pocket, "will you take this, just to help to—to help you over Christmas time"? She pressed a coin into the rough hand, and only the Angel knew that the coin had been saved for weeks with anxious care to supply a much-needed want of the nurse's own, and only she knew how very, very few more coins were left in that little battered purse.

"And I think," the nurse went on kindly, "that perhaps to-morrow a little bit of Christmas will come to your children, and a little Christmas dinner." For that thought whispered into her ear by the Angel had borne fruit. She remembered a friend who would certainly let her, to-morrow, take a dinner to these poor waifs of humanity; she remembered some toys treasured upstairs to be taken home on her next day off to her own little brothers and sisters, but which now she would give instead to these children, for whom otherwise no Christmas joy was in store.

In the corner of the ward the Angel stood silently looking down at the forlorn scrap of humanity with pitying eyes.

"I can bring him the best Christmas gifts," she whispered. Then the Angel stooped over the little cot, and kissed the child's tired eyes. And all at once a wonderful smile spread over little Joey's careworn face. He stretched out his arms to the Angel, and laughed softly. The nurse, who had returned and stood on the other side of his cot, thought that the child was delirious; but Joey could see what was invisible to her eyes.

"Happy Christmas," he murmured, "Happy Christmas!" and with that wonderful smile still upon his face his eyes closed, and the nurses whispered to one another that poor little Joey was dead.

But the Christmas Angel smiled, as she passed on her way. She knew that she had brought to this unloved waif, to this pitiful shred of humanity, her beautiful gift—a Happy Christmas.

### An Unexpected Meeting.

On the edge of a smoky Midland town, with its forest of tall chimneys, stands the hospital, its many windows brightly lighted. As the Angel enters she sees a nurse in spotless uniform moving down the corridor, and with her she passes through the door of a gaily-decorated ward, in which are three long windows on each side, with crimson blinds, and beyond, a door leading off to baths and lavatories. On the pale blue painted walls hang *Graphic* pictures, and on the beds are cosy crimson quilts. At either end a fire burns brightly, and in the middle of the well-scrubbed floor is a narrow table, covered by a snowy cloth, on which are plants and flowers. Near to this is a dressing-waggon with rubber wheels, to move quietly from bed to bed, holding all needful dressings for the wounds; another table holding testing apparatus, and various bowls and instruments, is close at hand. Between each bed is a highly-polished brown locker, which serves as a seat, and in which the patients keep their odds and ends. Two long settles by the fires, a medicine cupboard under lock and key, some screens covered with turkey-red, and a couple of chairs on wheels complete the furniture of the ward, which is No. 1 men's surgical.

There are no desperate cases in just now, all are mending steadily, and appear as bright and cheerful as surgicals generally are who have turned "Recovery Corner." One bed only is untenanted, but ready with its big macintosh and grey blankets for the next helpless comer. Stunted toilers in the depths of the earth are some of these men; others brawny fellows, used to the melting heat of furnaces, where they work stripped naked to the waist, with the sweat pouring off them; rough of speech and habit, but minding their "manners" in here to please the stately, kind-eyed Sister who rules over them, and who stands now, with Nurse Mollie, the "pro.," putting the last finishing touches to the flags and evergreens. These are regarded with serene satisfaction by the inmates, who have helped to rope the greenery hanging in festoons from the beams and round the doors and windows. Two men are up with broken arms, and Weston, who has lost a leg, is Nurse Mollie's useful man.

Amongst those in bed is Bevis, with his fractured ribs encased in strapping, dreading the day when it will be stripped off, although he knows how deftly Sister does it. But he tries to turn his mind away from disagreeable thoughts, and is picturing to himself the town outside, the crowded streets, and in the stone-paved market square the flaring naphtha lights, revealing various stalls, and many buxom good-wives, with shawls over their heads, buying provisions for the morrow.

Another occupant of a bed is a small man, "Dan," much "cockered" by his wife at home, greatly scorning the hospital provisions because he is in the "vittles" line himself. He hides his damaged leg under a big cage, which makes the graceful draping of his quilt a difficulty.

Then in the corner is sweet old Ben, bearing his dislocated thigh with much patience.

"Th' owd ooman has'na sent th' eggs her promised," he tells Nurse Mollie, when she makes him comfortable for the night.

"Oh! she's sure to come to-morrow," says the kindly pro., "and I'll find you a nice one for the morning;" and as she moves away the old man smiles, and says she is a "gradely lass."

Presently he hears the ward door open, and sees a tiny boy steps in, who says,

"A've coom to show 'ma cloathes," and turns round slowly for inspection. "The nurse hur maade 'em," he adds triumphantly, and well he may be proud, for they are of bright green damask, part of an old curtain, used for want of better materials. When he has received sufficient admiration, Nurse Mollie gets her banjo and treats him to some nigger songs.

The three men who are up now depart to a concert in No. 4. The Angel follows them. In a ward similar to the one just left, and as gaily decorated, are gathered all available patients, excited and happy. Though the hour is early, the entertainers are in evening dress, much to the delight of the women, to whom the fine clothes will furnish a topic of conversation for many a long day.

The Angel proceeds. She knows of others lying sad and lonely, and goes to whisper pleasant thoughts to these. Behind a screen is a young girl so badly burned that she cannot last the night. The Angel bends to kiss her, and the grief-stricken parents watching by wonder to see so sweet a smile suddenly pass over the poor child's face.

And now along the corridor there comes the steady tramp of feet, and Sister quietly says, "A case for us, I think," and, moving to the accident bed in No. 1 stands ready. The Angel in pity moves towards her. None but she notes the sudden pallor that comes upon the Sister's face as, in the man lying prone on the stretcher, she recognises the husband who deserted her eight years ago. A moment's deadly faintness, and then she bravely does her work.

Behind the screen the surgeons view the poor crushed form, and shake their heads, "No hope," they say, "too badly hurt for that," and then they leave him. She sinks upon the locker, gazing at the poor pale face before her; the blue eyes opened wide, unconscious of her presence. Then when Nurse Mollie comes to take her place she gently pushes her away. "Leave me alone here," she begs, and still she sits and hears the gasping moan she knows too well. Past years rise up before her—first, his careless ways; later, his idle disposition; and finally, the last sad, bitter quarrel before he left her. The distant music ceases, the lights go low, and all is quiet. At last he murmurs hoarsely, "Water!" and as she holds it to his lips, his eyes, suddenly conscious, rest upon her face, and she feels that he knows her now.

"Liz—can—you—forgive?" he gasps; and while she stoops to give the kiss which his eyes implore, with one last deep-drawn sigh he passes into the Silent Land. The Angel, as she spreads her wings to fly, looks back with pitying glance upon the woman, who, the tears dropping gently on to her clasped hands, kneels beside the narrow bed to pray that He, who once forgave a dying thief, will extend His mercy to yet another of His erring children.

### A Husband and Wife.

The city is picturesquely situated amidst somewhat lofty hills, but lies in a hollow, and the hospital which the Angel next visited is in its lowest part, close to far-famed hot springs.

The ward which she entered seemed at the first glance to be deserted by its occupants. Loving hands had decked the windows with evergreens, tasteful mottoes adorned the walls; while through an open door on the farther side the streaming light and sound of cheerful voices indicated that the patients were sufficiently well to have been dressed, and were now in the day-room beyond, enjoying the Christmas festivities provided for them.

A second glance showed that one patient still remained in the ward. He was a young man, and his pale face wore an earnest, thoughtful expression. He was dressed, and was being helped into a wheel-chair by a gentle-looking nurse, at the sight of whom the Angel smiled; for she had the face of one who has known sorrow and learned patience.

"Come," she was saying kindly, "you look a little more cheerful to-day. That is right, you mustn't worry, for really you are improving wonderfully."

The young fellow gazed at her gratefully. "Thank you, nurse," he said. "I do feel more cheerful, for I am looking forward so much to seeing my wife this evening. Yet I cannot help feeling anxious, too, for she is to tell me when she comes whether my situation is being kept open for me, or whether I shall go out from here to find myself once more among the unemployed."

"Have you been much among the unemployed, then?" the nurse asked, sympathetically.

"Indeed I have," he replied sadly. "Oh! nurse, I wish my parents had had me taught some good, honest trade, instead of making me a clerk. But now that I have at last got a good situation, aye, and got married on the strength of it, to get rheumatic fever and be laid up like this, seems very hard. It is my right hand I worry about most, for with that crippled I should be helpless indeed. But the baths have done it a lot of good, and your massage. You have been so good to me, nurse."

"Not at all," replied the nurse, kindly. "He whose birthday we celebrate to-morrow has said, 'Inasmuch as ye have done it unto one of the least of these my brethren, ye have done it unto Me.' And I am glad to be able to spend my life in His service."

The young man regarded her wistfully. "But am I even one of the least of His brethren?" he asked her. "I haven't thought much about Him, nurse, until I came here. The world has been very hard to me—it has been one long struggle. But here I have had nothing to do but lie and think, and everyone has been so good to me. It seems as if God must be good, after all, when His creatures are so kind."

The nurse looked pleased, and was about to reply, when the outer door of the ward opened to admit a young woman, neatly but plainly dressed, with a fair, pretty face, who stood hesitating on the threshold.

"It is my wife," said the young man.

"I will leave you, then," replied the nurse. "The patients are going to sing some Christmas hymns in the day-room, and want me to play for them. And you will like to see your wife alone, won't you?" Without waiting for a reply she was gone. And the Angel smiled again at the foolishness of mortals who ever think themselves alone.

But surely no Angel ever saw a happier meeting than that between these wedded lovers.

"My darling! to come this long way to see me! And are you very cold and tired?" the young man asked her tenderly.

"Not a bit, Jack, darling," the girl replied. "It is so good to see you again, and oh! how much better you are looking! It is all right about your situation; they are keeping it open for you. I went to see Mr. Mason myself; he seemed doubtful at first, but when I told him you were here he said that you were in the best possible place to get well quickly, and he would wait for you until you were strong again."

"Oh, how thankful I am!" the young man exclaimed. "I shall get well quickly now. See, my hand is getting quite strong again, and my ankles are much better, though I am not able to walk much yet. But they hope that I shall be able to come out in a few days, and we shall begin the New Year together, darling."

The little wife looked pleased, then her sweet face sobered. "Jack," she said, "I have been thinking, whatever should we do without hospitals? Why, in this illness of yours we shouldn't have had the money to get you the nourishment you needed, to say nothing of the treatment. What could we have done?"

Her husband looked grave. "Yes," he said, "it is very true. And they have all been so good to me here." He paused, hesitated, then with an effort spoke again. "Alice," he said, "I have been thinking too. I cannot help feeling that I have been too much occupied with getting on in this world, and have forgotten God. He has been very good to us, darling, he has given us to each other, and is giving me health and strength again. Shall we try, in the new life we shall begin with the New Year, to serve Him better by serving others?"

His wife looked up brightly. "Oh, I should so like to," she said, "especially to help the hospitals, shouldn't you Jack?"

"I should, indeed, he replied. "But now, they are going to sing in the day-room, and I should like to join them, if you will push me in." (And Alice wondered why the chair ran so lightly, for she did not know that the Angel was helping her.)

The day-room was a pretty sight. Even more tastefully decorated than the ward—the bright lights and happy faces made it seem like Christmas time indeed.

"Why, Jack!" exclaimed Alice. "I never thought a hospital was like this!"

"Neither did I until I came here," her husband replied. "But let me introduce you to Nurse Gertrude, who has done everything for me. You have her to thank for my life, darling."

And Alice, her eyes swimming in grateful tears, did thank her: and then Nurse Gertrude introduced her in turn to a little boy, the pet patient of the ward, who was going to hang up his stocking when he went to bed, and had every confidence that Santa Claus would not forget him. Then she asked Alice to join them at the piano.

"It is so nice," she said, "that we have no one in the ward very ill this Christmas, and so we can have some singing. They all do enjoy it so." And the Angel listened with pleasure as the glad notes filled the room—

"*Hark? the herald angels sing,*
*Glory to the new-born King.*"

### A Hero in an Accident Ward.

Without in the wide road that cuts through the slums of the East End of London there was noise and bustle and merriment. Even the very poor rejoiced, for it was Christmas Eve, and hard-earned pence were willingly spent on bits of holly and evergreen to show their gladness in the coming day. The naphtha lamps on the stalls flared on the eager faces and lighted up the busy street.

In the midst of all the traffic and din the great hospital kept watch and ward, its gates open day and night for the relief of pain. Sickness and accident know not time nor season, and on this night the hospital was as busy as the outside world.

In one of the great accident wards the approach of Christmas was evident. Holly-framed greetings decked the walls, and trails of ivy wreathed the archways. From the central lobby came the hum of voices, for the work of wreath-twining and motto-making was going on apace. Nurses, doctors, and dressers, all laboured willingly, and convalescent patients ran hither and thither cutting up evergreens and helping wherever they could. Everyone was doing his or her best to make Christmas a happy time for all who were compelled to spend the day far from those nearest and dearest to them. Some few, indeed, were homeless, and rejoiced to think that their Christmas would be passed in the midst of warmth and plenty, with goodwill and loving sympathy on every side.

As the Angel entered one of the divisions of the great ward there was a subdued sound of voices, for in the far corner a bandaged figure, which had tossed and moaned for hours, now slept quietly, a nurse keeping watch at his side. There is always this pitiful intermingling of joy and sorrow in hospital life.

Round the fire sat a group of men, all flying signals of distress in the form of bandages, slings, or crutches, but all looking bright and hopeful.

"They're wonderful places, is 'orspitals," said the young man in No. 12 bed, which was next the fire. "I don't know wot us poor folks would do without them."

"You may well say that," said Daddy 7, a toil-worn Deep-Sea fisherman. "I 'eerd the doctor say on'y this morning as you wos a credit to the 'orspital."

"I am allus glad," continued No. 12, "as I paid reg'lar to the 'Orspital Saturday Fund. I'd feel dreadful-like, lyin' 'ere and 'avin' everythink done for me, and me not able to feel as I 'ad done a bit myself to help keep the 'orspital up."

"I never thought of that," said one of the others, slowly. "It seemed so little what I could give."

"Every little 'elps," said No. 12, bravely.

"Tell us about your accident, George," said Daddy 7, who fathered every one in the ward, nurses included. As he spoke he drew his chair nearer to the bed. "I've allus wanted to 'ear about it. You've bin 'ere a long time, ain't ye?"

"Close on two months," said George. "I 'ad 'oped to be out for Christmas. My old mother was set on it, so I asked the doctor to try and get me well in time. 'You must buck up, my lad,' says he, 'and get on a lot quicker than you are doing, or it's a 'Appy New Year we shall 'ave to wish you 'ere,' says he. And it's right he was, for it will be another two or three weeks yet afore I can get up on crutches. It do seem 'ard sometimes to be lying 'ere so 'elpless," sighed No. 12.

"'Ow did it 'appen?" asked Daddy again, and George began his story.

"It was like this," said he. "You know I works at Gray and White's, the big stationers. I'm one of their packers. I've bin there ever since I left school, just bringing 'ome a few shillins a week to 'elp mother along at first, but now makin' a tidy wage with a bit to put by. And the best of it is," said George, warming to his subject, "as mother used to do office-cleaning in the City in them old days, but now she don't need to go to work at all. She stuck by me when I was 'elpless, so now I sticks by 'er; and that's fair, says I."

"Right you are," said Daddy, admiringly.

"And then there's Nelly now. She's my sweetheart, and we was goin' to be married this Christmas if it 'adn't bin for my accident. You know Nelly, don't you, Dad?"

"A fine lass," said Daddy; and George smiled gratefully.

"Nelly works at box-making. It's a poor job for a woman, long hours and little pay. But she won't be at that for long now."

"Wot about the haccident?" reminded Daddy.

"I'm just gettin' to that. Nelly 'as a young brother Billy, and when 'e left school, Nelly, she looked to me to 'elp 'im to work. I recommended 'im to our manager, and he got took on at the warehouse. We were all pleased at that, for so I could keep an eye on him. He's a nice lad is Billy, but a bit larky.

"Well, one day as I was goin' along with a big packing-case on my shoulder, I was passin' near by the lift in the warehouse, and I sees Billy and another little chap a-larkin' together. They keeps on 'ittin' one another in fun, and just as I passes, Billy 'e 'its the other chap one on the nob and then runs away. But he slips and falls, and rolls towards the lift. Then I sees as the gate were open and the lift movin', and there were Billy, 'eading towards the shaft. I turned sick and cold wi' fear, but it passed orf, and then out I rushed and grabbed 'im just in time. Then some'ow or another in my 'urry I slipped, and afore I could save myself my leg was caught and jammed in the lift."

Daddy gave a long-drawn breath.

"'Twas bad," said George, "for they do say as the bones were stickin' through when they picked me up. But I fainted away, and all I remembers was sayin' to the doctor arter I was brought 'ere, 'Syve me leg, mister,' and then I went orf agen.

"They tell me they thought the leg would 'ave 'ad to be cut orf, but they took me into the theayter, and give me ether; and they sawed and chipped and fixed the bones up with wire, so they say, and then I come back to the ward.

"When I come to there was the doctor and the nurse standing by the bed, and 'e says, 'We've saved the leg, my lad.' 'Thank God,' says I; and then I cried a bit, for I felt as weak as a baby.

"That was two months ago, and 'ere I am, and the leg 'ealin' beautiful." And No. 12 looked admiringly at the limb as it swung in the cradle.

"And you 'urt yerself a-tryin' to save the little chap," said Daddy. "Wy, you're a 'ero, George; that's wot you are."

But George looked troubled. "I couldn't 'ave let the little chap get 'urt, and 'im Nelly's brother too."

But the Angel smiled very gently on the pair. She agreed with Daddy 7.

### JIM'S CHRISTMAS PRESENT.

The Angel breathed a sigh of relief as she spread her wings, and soaring in the air soon left behind the grimy metropolis with its murky sky and all its sights and sounds of misery and pain, which could not fail to wound her in spite of the cheering incidents of love and tenderness she had just witnessed. Her path now led her to one of England's warmest corners, and after the snow and frost, she hailed with delight the sheltered pine forests of the southern coast. Pausing here, she made her way to a long, low, stone building, every window of which (and they were many), in spite of the time of the year, stood widely open, as though inviting her to enter. The brilliant moonlight streaming in through the open windows was unobstructed by curtains or window drapery, whilst the glowing fires in the wards gave them a comfortable and homely look as the flickering shadows played upon the walls.

Passing from ward to ward the Angel could scarcely believe that the Institution was devoted to consumptive patients, and as her glance fell upon the sleepers around her she thought she must have mistaken her goal. Where were the pale and emaciated men and women? Where the weary sufferers torn in pieces by the hacking cough, tossing from side to side, burnt up by fever, and drenched in sweat, wishing vainly for the daylight? Instead, she beheld peaceful sleepers whose faces tanned by exposure to sun and air, spoke eloquently of renewed hopes and returning health, their well-nourished frames showing unmistakable signs of good food and powers of assimilation. As the Angel moved wonderingly through the building, voices in one of the smaller wards reached her ear, and, thinking that perhaps she might learn the why and wherefore of all these changes, she made her way in their direction.

Following the sound, she found herself in a comfortable room containing three beds, each provided with a screen of moveable white dimity curtains; the patients, instead of being asleep, were exchanging confidences in the firelight. The voice of one of them seemed strangely familiar, and the astonishment of the angel was great when she recognised the speaker as a little servant girl to whom she had been sent some eight months before with a message of hope and love when the girl was lying in one of the beds of a big London chest hospital, recovering from the fright and ill-effects of a sudden attack of hæmorrhage. Poor child, she was barely seventeen, and her life had been a hard one, although the story is common enough among her class. The eldest of a large family, her father having died of consumption, she had been sent to earn her own living directly she had "passed her standards" at the early age of thirteen. Never having had the chance of a decent start in life, she could only take the roughest of "places," and at the time of her break-down was the cruelly over-worked and underfed "slavey" of a third-rate London lodging-house. Up and down the weary stairs she toiled, generally with heavy loads, from the first thing in the morning to the last at night. Kind words were practically unknown to her, the food was poor and scanty, and her wretched wages being insufficient for even the cheapest of respectable-looking top clothing, what wonder was it that the few under-garments she possessed were of the poorest and thinnest description? and that her boots were so broken and decayed that it was only with the greatest difficulty she could keep them on her feet at all. It was, therefore, not surprising that, running errands in her half-starved and slipshod condition, she caught a dreadful cold when sent out in the snow one day; the cold developed into a "cruel corf," and she grew thinner every day. At last came the crowning catastrophe of an attack of hæmorrhage, and her frightened mistress had her promptly removed to the "'orspital," where the Angel had last seen her.

As the Heavenly Visitor drew near she heard little Mary trying to cheer up one of her companions, who was crying. "Come, Annie," she said, "don't fret yerself so, h'I know as 'ow its 'ard when yer first come in, yer feels so lonely, but yer'll soon see 'ow 'eavenly it is to 'ave nothing to do but lay out o' doors and 'ave plenty to h'eat, same as if yer was a lidy."

"T'aint that," Annie replied, between her sobs. "It's Jim, the young man as h'I'm akeeping company with. When I come in 'ere mother said as 'ow h'I'd 'ave to break off with 'im, but h'I don't want to—'e's a soldier, 'e h'is."

The little comforter lay quite still turning this over in her mind, when suddenly the third occupant of the room, a woman of five-and-twenty, with a sweet, patient-looking face, raised herself on her elbow and said, "Well, you are a little goose, Annie; fancy going on like that, you ought to be downright ashamed of yourself, anyone would think you hadn't a scrap of sense. You have only been here less than a week, and I heard the nurse saying you weren't likely to gain weight or do any good as long as you keep on fretting, and she thought the doctor very likely wouldn't let you stop unless you began to pick up a bit." The poor culprit sobbed more bitterly still, and gasped out something that sounded like "trying."

"Trying, are you," said the older woman; "I'd turn over a new leaf and try to some purpose, if I were you. How do you think you are ever going to get better crying at meal-times and even making your milk watery? When you go home you'll be so much worse Jim 'll give you up, and then you'll be no better off. Now, if you'll only be sensible and behave yourself when your time's up and you go home, Jim will see for himself how you are getting on, and there'll be no need to cry and bother yourself at all."

Annie stopped crying, and sat up in bed eagerly. "Oh, that would be too good!" she exclaimed.

"Well," replied her friend; "it's your own fault if you don't; you know the doctors told you that your lungs weren't very bad, and you ought to grow quite strong and well. Now, to-morrow is Christmas Day; just you make a Christmas present of yourself to Jim, and begin by drinking all your eight mugs of milk and eating just four times as much as you always do. Look at me, I put on a stone in less than a month, and the doctor says I'm on the road to being cured. Want to know how I did it? Why, when I didn't feel as if I could manage the beef and mutton and so much milk, I just said to myself, remember you've got to be cured to go back to the children and Tom (he's my husband), and at last it seemed to grow easier, and as one feels how much better and stronger one gets one don't need to worry, but just think how glad they will all be to have you home again quite strong and well. Now you begin to-morrow, there's a good child, and do just as I tell you, drink your half-pint before you get up, eat an enormous breakfast, and after prayers don't dawdle and wait for nurse to hunt you out, but get a rug and hot-water bottle, and I'll choose us a nice sunny spot where we can have our lounge chairs side by side and have a jolly Christmas together, thinking about next year, when we'll both be strong and well again. Christmas Day will be grand for a start; you'll find you can eat turkey and plum pudding as well as anyone, and in the evening there's to be a Christmas tree and carols and music, so we shan't any of us feel home-sick. Do you think I don't often want my babies and my good man? But I know if I fret I shan't ever get well; so, please God, I'm making the most of my chance."

Annie had by now quite ceased sobbing, and she promised to try and do her best in the future. Presently one by one they all fell asleep, and the Angel suddenly realised why she had been sent to them. As she hovered there unseen, she whispered such sweet messages of courage, hope, and love to the three sleepers, and drew such dream pictures of the pleasures of renewed health, that each felt a fresh access of strength with which to continue the long, uphill fight against the demons of death and disease lurking even in their very midst.

### Susie's Vision.

Christmas Eve once more, or rather Christmas Day, for the chimes of St. Jude's have announced midnight, and now the bells are ringing out a peal so wild and exultant that they seem to tell not only of peace and goodwill to men, but, as mystics of "le moyen âge" believed, to bid defiance to all the powers of darkness. In the shadow of the church stands a little band of singers, nothing daunted by the clamour of the bells, singing with somewhat raucous voices an old-fashioned carol, chiefly remarkable for its many verses and doubtful theology. As soon as it is over, with just a pause for breath and a general clearing of throats, they walk a few yards further on, and halt before the grey stone steps of the county hospital, and with a view to enlivening the inmates, burst with all their might and main into what they consider the most appropriate selection their *répertoire* contains, "God rest ye, merry gentlemen."

The bright red light over the hospital door throws quite a radiance into the misty gloom; it suggests cheerfulness and warmth, and puts into shade the cold yellow flame of the street lamps. Within all is cheerful too, the hall is gaily decorated with holly and ivy, and a gigantic "Welcome" in red letters on a frosted white ground gives greeting to all comers.

Newcome Ward, after a day of rush and hard work, is peacefully quiet. At all times it prides itself upon its well-deserved reputation for smartness and cleanliness, but to-night one would imagine that fairies (if those little people do not migrate for the winter) had been busy making Newcome the very prettiest and daintiest ward possible. The clock over the door is wreathed with holly and mistletoe, and above the clock is a "Gloria" in letters large enough to be seen by Granny Tozer in the last bed at the other end of the ward. Festoons of evergreens adorned the walls in spite of the house surgeon's murmurs of "germs" and "dust." Tradition counts for much, especially at Christmas time, and Sister pleaded so pathetically, and promised so decidedly that they should come down as soon as Christmas festivities were over, that, as usual, she had her own way in the matter. Flags are much in evidence, and the picture-frames have had an extra polish to mark the season. Small wonder that Sister, staff nurse, and pros. have gone late to bed with weary arms and aching feet. In Sister's sitting-room adjoining the ward are piles of useful and pretty things destined to give great joy to their recipients, and a whole crowd of toys sent by kind friends for the children. Night Sister and nurse are waiting till all are sound asleep to go round and fill the stocking which hangs on each youngster's bed with sweets, oranges, crackers—to be crowned by toys chosen with due regard to the tastes of the stocking's owner.

The ward is very still when the Angel enters and stands unseen beside each sleeping form. Baby Billy occupies the cot; he is more than convalescent, but cannot be spared by the nurses, who adore him, until Christmas is over; he sleeps tranquilly, a picturesque little figure, both chubby fists thrust amongst tumbled golden curls. In No. 28 bed lies someone still awake. It is Susie—her thin, long arms tossed over her head—with flushed cheeks, and heart throbbing almost painfully in anticipation of delights to come. The Angel remains there by the little girl's bed, and on her radiant face is a look in which all love seems gathered, as to her gaze is open each thought of Susie's mind.

Poor Susie! In her seven years of life she has known no joy, and now suffering has brought her happiness, for in the hospital she has found love and kindness and all things which make the joy of living. Lizzie in the next bed has a diseased hip, and pays Newcome ward periodical visits; she has spent Christmas there before, and has been feeding Susie's imagination with tales of the treats in store. Susie has never before thought of Christmas, except as a time of year when the weather was unpleasantly cold, and when father and mother were sure to be very drunk and treat her even more unkindly than usual. Though she is their only child, for the last year she has gone out "cleaning" for twopence a day, and that small sum has invariably been confiscated by the mother, who sometimes pawns all their belongings for drink. A fortnight ago, on a bitterly cold night, her parents had a fight—a not unusual occurrence—and the man, in his drunken fury, turned both wife and child out of doors in their thin nightgowns, and there they stayed on the doorstep till morning. Pneumonia was the result for Susie, and she would have died at home through sheer neglect had not a rough but kindly neighbour urged the mother to take her child to the hospital, threatening to make the street "too hot to hold" her if she refused.

From Lizzie's description, Susie has a vivid idea of what Christmas Day will be like, and her restless fancy is now picturing for the hundredth time every event of that eventful day.

First of all, when they wake in the morning the now empty stockings will be full, and there will be the bliss of taking out their contents, and exclaiming over each surprise. Susie has all day had a nervous fear that Santa Claus will forget her, as her bed is in the corner, so she has borrowed one of Granny Tozer's grey worsted stockings as being more likely to attract his attention than her own little ragged one, and it hangs conspicuously at her bed head, to her great contentment. She hopes fervently that Santa Claus will bring her a fairy tale book like the one he gave Lizzie last year, though she has not voiced her wish again since Mrs. Hunter, in the opposite bed, said she didn't "hold with fairies and sechlike folk, they bain't respectable." A doll is the height of Susie's ambition, a doll of her very own, with yellow hair and blue eyes, such a doll as, though she little knows it, is in Sister's room at this minute, and whose destination is the grey worsted stocking.

Next will come the nurses to sing a hymn about the Christ Child and shepherds and angels. She half turns, and takes in her hot hand a card from the locker-top. It is a picture of the Holy Mother and Child with angels bending in adoration. The chaplain gave it to her yesterday when he tried to explain the story of the greatest love in the world. She asked what "them feathery things" were, and he told her of the angels who guard the ways of little children, and keep them from harm and danger.

"Ah," said Susie, with a sad look on her old face, that has never been young. "They don't come down our street."

Who will be able to eat on such a morning, when each grown-up patient will have at least one present brought to her with her breakfast, and all will be chattering and laughing and comparing gifts? And then Sister will come with a smiling "Merry Christmas to you all!" and later on the doctors will look in and nod and smile to everyone. The matron will walk round the ward, and have a little chat with each patient and admire the presents. Dinner comes next—not the every-day meat and pudding, good as they are, but a big turkey carved on the ward table where all can behold it, and pheasants besides, and a Christmas pudding—the children will hold their breath as the Senior Honorary with due deliberation applies a lighted match to that mysterious pudding, which suddenly is wreathed in flame. Susie smiles at the tale of Teddy, who last Christmas cried loud and long because he thought the pudding would be burned up and he wouldn't get any, and then resumes her waking dream. In the afternoon ladies and gentlemen and prettily dressed children will visit the ward, and then it will be tea time—cake as well as bread and butter—and soon after tea—oh, dear delight!—a wonderful, and beautiful, and dazzling thing she can only dimly conceive—the Christmas tree—and Susie gives a big sigh, in which are mingled bliss that such happiness may be hers and longing for the delectable moment to arrive.

Then the Angel, knowing how much Susie will need all her strength for the due enjoyment of the morrow, bends over the child's bed in tender benediction before she vanishes. A sudden sense of peace and restfulness steals into Susie's mind, and with a gentle smile she falls asleep.

Good night, dear child; sweet dreams be yours, and a yet more happy waking!

### THE ANGEL'S APPEAL.

The Angel had finished one part of her mission. She had seen, with her own eyes, what was being done in the hospitals for the relief of suffering humanity. She had heard, with her own ears, the testimony of all sorts of people, under varying conditions and different circumstances, as to the benefits they had derived from the ministrations of skilful doctors and capable nurses. It remained for her to turn to the best practical account, on Christmas Day, the knowledge she had gained on Christmas Eve. This was what she now set herself to do.

And first she sped on her way to the homes of the poor, of those whom she knew had little to give, but were yet able, by reason of their vast number, to render valuable support to the institutions in which she had seen so many of them tenderly cared for. Nor, if some turned a deaf ear to her gracious monitions, were others unresponsive. She learned that there were men who, mindful of the manner in which a son injured by an accident, or a daughter suffering from a wasting disease, had been treated in the "'orspital," frequently dropped their pennies into the box outside the building on their way to work; and women who, out of the scanty sum supplied to them for housekeeping purposes, now and again cast their mite into the same treasury box of the Master. And there were those who, with no personal experience of the benefits of the charities, were moved by her recital of the touching story of George, or the account of Susie's vision, to promise to spare in the season of Christmas festivity a piece of silver towards the maintenance of the great homes whose doors stood open day and night for the reception of the sick and suffering. When the Angel had reminded the multitude who cannot afford to pay for medical advice or proper nursing—those from among whom the vast majority of patients in the hospitals are drawn—of their obligations, she passed on to the people who, although in a position at the moment to provide the doctor's fee, and to summon the professional nurse, may, in case of graver illness, find themselves compelled to seek admission to the charitable institutions which they too often complain are of "no advantage to them." Nor in vain did she relate in middle-class dwellings, where the income of the bread-winner is precarious, the experiences of the young clerk whose chances of recovery in his own home would have been slender indeed; while some, who had just been restored to health by the skilful attendance they had been able to command, joyfully welcomed the suggestion to commemorate the event by assisting a hospital in which their less-favoured brothers and sisters could be treated for a similar disease.

And thus, changing her plea according to circumstances, she proceeded to make her way into the mansions of the rich. Here, she knew, eminent physicians were consulted in cases of illness regardless of cost, and trained nurses engaged as a matter of course. Here were the people who thought nothing at Christmas of spending a five-pound note upon a child's toy, or of giving a sovereign to a club waiter. From these homes of ease and comfort she was aware the hospitals expected the substantial sums which justify them in making improvements and enlargements—in augmenting their accommodation for the ever-increasing crowd of applicants for vacant beds. Often, she knew, they did not expect in vain; but she knew, too, of the many who, with every possible luxury at their disposal, and with power to secure for their afflicted ones all the known alleviations of pain, and command all the resources of human science, never troubled themselves about the thousands of fellow-creatures who but for the hospitals would not have the remotest chance of being healed in the event of an injury, or cured in the case of attack from an insidious disease. With these, on the morning of Christmas Day, she pleaded, urging them not to neglect claims which were recognised and endorsed in the sphere from which she had descended; and her parting injunction, as the joy bells called all classes to celebrate the Festival of the Incarnation, was an echo of her Lord's own words—"Freely ye have received, freely give."

### THE "KIDDIES'" CHRISTMAS.

(*Suitable for Recitation.*)

I'm in the 'orspital, yer know,
I'm number twenty-nine;
I hev to lie right on my back
'Cos of a "curis" spine.

An' Christmas time 'll soon be here,

I wants right bad to stay,

If Sister 'll beg house doctor hard

He 'll not send me away.

They allers tries to get 'em 'ome,
All them wot's fit to go,
An' only takes bad cases in,
The wards is lighter so.

Poor Tommy Brown, next cot to me,
Was in last Christmas time,
An' helped the nurses trim the wards,
An' made some festoons, prime.

The students an' the nurses fetch
An' bring in heaps o' green,
An' hangs festoons, an' flags, an' wreaths,
Tom says, an' Tom he's seen.

They don't take no "off dooty" time,
They're in the wards all day,
You see they've all their work to do,
As well as all the play.

They gives us flow'rs and things to make,
So's all on us can say,
We done the decorations too,
All smart for Christmas Day.

On Christmas Day, when we wakes up,
There's letters by our cots,
An' all on us has Christmas cards—
Some o' the kids 'as lots.

Then nurses comes an' sings to us,
They goes to ev'ry floor,
A singing "Herald Angels" hymns
And carols at the door.

The dinner-table's trimmed wi' flow'rs
An' coloured paper lace;
There's ginger beer an' lemonade,
An' all on us sings grace.

There's doctors carves in ev'ry ward,
And ladies hand's our plates;
We've crackers, sweets, an' oranges,
Plum puddings, nuts, an' dates.

Then ev'ryone wot's well enough
Round all the wards can go;
Last year Tom's doctor carried him,
He's too ill now to know.

An' after tea there's Christmas trees,
An' toys on with our names;
There's concerts, lanterns, play-actin',
An' lots o' other games.

All round the tree's a ring o' cots,
An' visitors outside,
And patients from the other wards,
An' lots o' folks beside.

The doctors climbs up ladderses
To cut the presents' strings;
The patients wot's too ill to come
Their nurses takes their things.

But they can't see the 'lectric light
Wot lights the tree, nor yet
They never know wot funny things
Nurses an' doctors get.

But this year pore old Tom won't see
Our tree, an' have his toys;
They moved him to the private ward,
So's not to hear our noise.

"Pore Tom's gone home," our Sister says,
"Last night the Angels come;"
But Heaven's where Sister means, I guess,
'Cos Tom ain't got no home.

But Bess says Angels have a tree
(An' Tom's a Angel now);
It's all lit up with shinin' stars,
An' moons on ev'ry bough.

The presents off the Angels' tree
Can't never spoil nor break;
In Heav'n no one don't have no pain,
Nor crooked backs wot ache.

An' nobody don't climb up steps
To reach the things up high;
They cuts their toys off all theirselves,
'Cos little Angels fly.

They've swords, an' guns, an' butchers' shops,
An' dolls, an' heaps o' things;
How Tommy'll manage, I don't know,
There ain't bin time for wings.

### A WORD IN SEASON.

The present war has probably knit the people of the Empire together more closely than ever before. Our Queen has set all classes a noble example in generosity, not only towards the war funds, but towards all the older charities which must be maintained whether we are at war or at peace. It is essential that our hospitals and kindred institutions shall receive the most liberal support at this time. Indeed, we are confident that those who give to the war funds, will regard such gifts as extra donations in addition to the sums which they have heretofore set apart year by year for the older charities. In giving to hospitals all should remember that they are indirectly giving to some extent to our soldiers and sailors, whose wives and families are mainly dependent upon the hospitals in cases of illness during their absence.

### SWEET CHARITY'S GUIDE TO CHRISTMAS GIVERS.

### GENERAL HOSPITALS.

**Charing Cross Hospital**, Agar Street, West Strand, W.C.—This institution is situated in the heart of the metropolis, and in some of its most densely-populated and poor districts. Surrounded by crowded thoroughfares, it has to provide for an unusual number of accidents. The hospital has a convalescent home, containing 50 beds, situated on Limpsfield Common. £15,000 a year is required annually from voluntary sources to maintain the hospital and the home. Secretary, Mr. A. E. Reade.

**Great Northern Central Hospital**, Holloway Road, N.—Letters of recommendation are not required at this hospital; 159 beds are now open. Over 2,000 in-patients are treated annually. The hospital is unendowed, and the reliable income is quite inadequate to meet the necessary expenditure—over £10,000 being required annually from voluntary sources. Special precautions are taken here to restrict the benefits of the charity to the sick poor. Secretary, Mr. Lewis H. Glenton-Kerr.

**Guy's Hospital**, London Bridge, S.E.—At the present time Guy's Hospital requires substantial support from the benevolent in order to bring it to the highest state of efficiency and usefulness, and £25,000 per annum must be obtained from voluntary sources to supply the deficiency in the income necessary for the maintenance of the complete establishment. Treasurer, Mr. H. Cosmo O. Bonsor, M.P. Superintendent, Dr. E. C. Perry.

**Hampstead Hospital**, Parliament Hill, N.W.—During 1898 there were 327 in-patients besides 318 minor accidents and casualties treated in the hospital. There were also 2,288 attendances in the out-patient department. It is estimated that the expenditure in 1899 will exceed the income by over £1,200. Additional funds are urgently needed to meet this deficiency. The present hospital being totally inadequate to meet the wants of the neighbourhood a building fund has been started for the erection of a new hospital. Hon. Secretary, Mr. R. A. Owthwaite.

**Italian Hospital**, Queen Square, Bloomsbury, W.C.—The old building having proved quite inadequate, and most unsuitable for the work which the hospital had to do, the committee are building a new hospital on the old site and a site adjoining. Funds are urgently needed for the erection of the new hospital. Secretary, M. G. Ferrari.

**King's College Hospital**, Portugal Street, Lincoln's Inn, W.C.—2,384 in and 23,930 out-patients were treated in 1898, and the expenditure exceeded the income by nearly £6,000. Warden, Rev. N. Bromley.

**London Homœopathic Hospital**, Great Ormond Street, W.C.—The annual expenditure at this hospital is at the rate of £9,500. The reliable income is about £6,500 per annum. There is, therefore, an urgent need of an immediate and substantial increase in the annual subscription list. Treasurer, Lord Cawdor, 7, Princes Gardens, S.W. Secretary-Superintendent, Mr. G. A. Cross.

**London Hospital**, Whitechapel Road, E.—This is the largest voluntary hospital in this country. From this fact, and from the zealous manner in which the work is carried on, we make no doubt that the public will give largely to its funds, in testimony of their appreciation of the enormous value of what is being done there for the poor of East London. It is in serious want of funds, as the committee depend on voluntary contributions for £40,000 a-year to enable them to maintain the 656 beds which are daily occupied by urgent cases. House Governor and Secretary, Mr. G. Q. Roberts, M.A.

**Metropolitan Hospital**, Kingsland Road, N.E.—The need for this hospital in the poor and densely-populated districts in the midst of which it is situated, is shown by the fact that both the in and out patients have been steadily on the increase for several years past, the numbers treated last year being, in-patients 962, and out-patients 30,017, the attendances of the latter numbering 99,330. There is a debt at the present time of over £4,500, incurred during the past four years. The charity is much crippled by lack of funds, and several beds have had to be closed, leaving only 70 now available for in-patients, although there is accommodation for 160. Secretary, Mr. C. H. Byers.

**Middlesex Hospital**, Mortimer Street, W.—The in-patients for last year numbered 3,607, and the out-patients 37,447. The income from all sources, including legacies, was £31,000, and the expenditure £36,000; deficiency on the ordinary income and expenditure, £6,000. The cancer wards are a distinguishing feature of the hospital, and their extra nursing, costly treatment, and unlimited dietary add largely to the expenses of the hospital. Towards the extension of this department, by removing it from the main body of the hospital and building a new wing, £9,000 is still needed. Secretary-Superintendent, Mr. F. Clare Melhado.

**North-West London Hospital**, Kentish Town Road, N.W., was founded in 1878, and is the only institution of the kind in the north-west district. It has 53 beds, and last year there were 601 in-patients and 18,033 out-patients. Secretary, Mr. Alfred Craske.

**Poplar Hospital for Accidents**, Blackwall, E.—Situated amongst a teeming population of poor hard-working people in a district which may be called the "workshop" as well as the "port" of London, this hospital is doing an excellent work. The demands on this institution of late years have greatly increased, and consequently further subscriptions and donations are very necessary. Chairman, the Hon. Sydney Holland. Secretary, Lieut.-Colonel Feneran.

**Royal Free Hospital**, Gray's Inn Road, W.C.—Having no endowment, this hospital is entirely dependent for support on the subscriptions of its governors and the voluntary donations and bequests of its friends. It admits into its wards over 2,000 poor sick persons annually, besides administering advice and medicine to more than 25,000 out-patients who resort to it, not only from the crowded courts and alleys in the immediate neighbourhood, but from all parts of London and the suburban districts. The relief thus afforded is effected at a cost of about £12,000 per annum. Secretary, Mr. Conrad W. Thies.

**St. George's Hospital**, Hyde Park Corner, S.W.—This institution contains 351 beds, and during 1897 treated 4,689 in-patients, 15,518 out-patients, 12,096 casualties, and 404 maternity cases. Secretary and Superintendent, Mr. C. L. Todd.

**St. Mary's Hospital**, Paddington, W.—Very heavy work is thrown upon this institution owing to the large area of scattered poor which it has to assist. It is not so well supported as it should be, in spite of, or rather because of, its many wealthy neighbours. The board of management, therefore, urgently appeal for further support. The special wants of the hospital are: (1) New annual subscriptions; (2) donations for the endowment of beds and cots. The hospital is free, and no urgent case is refused admission. Secretary, Mr. Thomas Ryan.

**St. Thomas's Hospital**, Westminster Bridge Road, S.E.—Since appealing to the public in 1895 the Governors have been able to reopen three of the five wards closed in 1871 for want of funds. It is, however, of great importance that the remaining two wards (at present used for paying patients)

should be rendered available for the sick poor of South London, for whom so little hospital accommodation is provided. During the last 15 years the population of the district has increased at the rate of about 25,000 annually, and yet there are fewer beds available now than in 1871. A Bed Endowment Fund has recently been established, to which contributions of £1,000 are invited. Treasurer, Mr. J. G. Wainwright.

**Seamen's Hospital Society, "Dreadnought,"** Greenwich, S.E.—During 1898 representatives of over 50 different nationalities weie admitted to the various establishments of the Society. The branch hospital at the Royal Victoria and Albert Docks is being enlarged, the accommodation there being inadequate to meet the requirements of that busy centre of shipping. The London School of Tropical Medicine, which is under the auspices of her Majesty's Government, is established at the branch hospital of the Society. Secretary, Mr. P. Michelli.

**University College Hospital,** Gower Street, W.C.—This hospital was founded in 1833, and during the past 66 years has treated over one million and a-half patients. In 1898 alone 2,922 in-patients and 46,846 out-patients were treated, 300 patients were sent to convalescent homes, and nearly 1,000 surgical and other appliances were supplied to poor patients. The financial condition of the charity is at all times a matter requiring the serious consideration of the committee, as the reliable income from all sources is only £7,500, and the necessary annual expenditure is over £18,000. Secretary, Mr. Newton H. Nixon.

**Westminster Hospital,** Broad Sanctuary, S.W.—Out of an expenditure of £17,000, less than £3,000 is assured to this charity, so that about £14,000 has to be made up each year in subscriptions, donations, and legacies. The work of the hospital in 1898 included the treatment of 2,637 in-patients, 23,991 out-patients and casualties, and 226 lying-in women. The hospital does an immense service to the country by training a large number of excellent nurses. Secretary, Mr. Sidney M. Quennell.

### PROVINCIAL HOSPITALS.

**The Birmingham General Hospital,** Founded 1766.—The new building of this hospital was opened by H.R.H. Princess Christian, on behalf of H.M. the Queen, on July 7th, 1897, and contains 340 beds. To meet the increased accommodation provided a larger income is necessary, and an earnest appeal is made for new and increased annual subscriptions. House Governor, Mr. Howard J. Collins.

**Bournemouth National Sanatorium for Consumption and Diseases of the Chest.**—Patients who are convalescent, who require further medical treatment and change of air, or who are suffering from an incipient form of the disease, are cases for the relief of which this institution was founded. There is accommodation ror 31 men and 31 women, and about 300 patients from all parts of the kingdom are treated annually. The committee earnestly solicit liberal support, as this national sanatorium is entirely dependent on voluntary contributions. Secretary, Mr. G. Lowe Riddett.

**West Ham Hospital,** Stratford, E. — During 1898, 744 in- and 22,530 out-patients, were treated. The income, which is derived from voluntary contributions, is quite inadequate to meet the demands of the hospital, which does admirable work in a very poor and densely populated East End district, remote from the observation of the wealthy. The hospital is at present £700 in debt to its bankers. Secretary, Mr. Leonard W. Rea.

### SPECIAL HOSPITALS.

### CONSUMPTION.

**Brompton Hospital for Consumption and Diseases of the Chest,** S.W.—This hospital is well known as one where patients are well cared for, and every effort is made to brighten their clouded lives. The committee appeal for new subscribers to replace those removed by death, and other causes, and for assistance to enable them to erect a much-needed country branch and a home for staff and private nurses on ground in rear of the hospital. Funds and gifts are also asked for towards the annual Christmas tree. Secretary, Mr. W. H. Theobald.

**City of London Hospital for Diseases of the Chest,** Victoria Park, E.—Situated in the East of London, where the disease it treats is so common, about 1,000 in-patients and 15,000 out-patients are treated yearly. 34 beds are still closed owing to ack of funds, leaving only about 100 available for patients. Assistance is much required to meet current expenditure. Secretary, Mr. Henry T. Dudley Ryder.

**North London Hospital for Consumption and Diseases of the Chest,** Mount Vernon, Hampstead, N.W., and Fitzroy Square, W.—The "open-air" treatment of consumption is carried out at this institution, which is admirably situated on the hills of Hampstead, some 400 feet above sea level. The results of the adoption of this method of treatment are so far most encouraging, and the committee are now erecting special balconies in order that the treatment may be extended. There are at present 80 beds in the hospital. Funds are needed for general purposes and for the building fund. Secretary, Mr. William J. Morton.

**Royal Hospital for Diseases of the Chest,** City Road, E.C.—The expenditure at this hospital exceeds £8,000, towards which there is an annual subscription list of some £2,250, and dividends amounting to £120. Funds are urgently needed. Donations will be gratefully acknowledged by the Secretary, Mr. John Harrold.

**Royal National Hospital for Consumption and Diseases of the Chest,** Ventnor, Isle of Wight.—A new block for 21 additional men patients was opened in 1899. As the expenses exceed the assured income by £4,000, the committee appeal for additional annual subscriptions and donations. Secretary, Mr. Ernest Morgan. London office, 34, Craven Street, Charing Cross, S.W.

### LYING-IN.

**City of London Lying-in Hospital,** City Road, E.C.—Funds are urgently needed to carry on the general work of the hospital, and also to defray the cost of building new dormitories, which the committee are now providing for the nurses and pupils at an outlay of over £3,000. Secretary, Mr. R. A. Owthwaite.

**Queen Charlotte's Lying-in Hospital,** Marylebone Road, N.W.—This hospital received 1,112 patients into its wards last year, and, in addition, attended 1,070 patients at their own homes. The enlargement and improvement of the hospital is now almost completed, and much-needed additional accommodation is now becoming available. The new nurses' home, which has been erected opposite the hospital, was opened by T.R.H. the Duke and Duchess of York in July last. Upwards of £7,000 is still needed for these works and for new furniture for the hospital and home. Donations to the building fund, as well as for general maintenance, will be thankfully received. Secretary, Mr. Arthur Watts.

### EPILEPSY AND PARALYSIS.

**National Hospital for the Paralysed and Epileptic** (Albany Memorial), Queen Square, W.C.—The total number of beds provided at this hospital and its Finchley branch is 200, but the accommodation is still painfully insufficient to meet the requirements, as the large majority of patients are unsuited to general hospitals. Many urgent cases are always waiting for beds. Besides the hospital for treatment, there is a pension fund for the incurables. The annual expenditure is £17,000, of which upwards of £10,000 must be raised in benefactions. Director, Mr. B. Burford Rawlings.

### CHILDREN.

**Alexandra Hospital for Children with Hip Disease**, Queen Square, Bloomsbury, W.C.—The necessity for this hospital for hip disease may best be shown by the fact that nearly three-fourths of its patients come from other London hospitals, the reason being that no general hospital can retain a patient for two or three years, as is not infrequently done in this hospital. £1,000 is urgently needed for maintenance, and £1,000 to complete the furnishing fund of the new hospital. Secretary, Mr. Stanley Smith.

**East London Hospital for Children and Dispensary for Women**, Shadwell, E.—It will readily be understood that the struggle for existence in such a neighbourhood as Shadwell is very great, and for this reason the committee of the East London Hospital for Children appeal to the residents in wealthier districts for funds to enable them to help those who are unable to help themselves. The seaside branch at Bognor (28 beds) for convalescent patients materially increases the usefulness of the charity, and at the same time its need of support. Secretary, Mr. Thomas Hayes.

**North-Eastern Hospital for Children**, Hackney Road, Shoreditch.—Founded 1867. This hospital is required to take an important part in the care of sick children of London's poor with very inadequate accommodation. It has at present only 57 beds, and ministers to a population of 500,000. Besides the ordinary patients there are over 70 accident and emergency cases every week, and it is necessary to refuse admission to the wards to between 600 and 700 urgent cases in the course of a year owing to want of room. A building scheme involving the expenditure of about £32,000 has been entered into by the committee, who have thus far succeeded in obtaining £12,500 towards the amount. Further help is urgently needed. Secretary, Mr. T. Glenton-Kerr.

**Paddington Green Children's Hospital.**—This hospital was established in 1883. It was rebuilt and enlarged in 1895. It provides 45 cots. There is a large out-patient department, where over 1,000 patients are treated every week. Funds are urgently needed for the maintenance of both the hospital and the home. The hospital owes its bankers £1,000. Secretary, Mr. W. H. Pearce.

**The Hospital for Sick Children**, Great Ormond Street, London, W.C.—The Hospital for Sick Children is the oldest and largest children's hospital in the British Empire, and needs £1,000 a month to keep open its wards, and so treat about 2,000 in-patients a year. New subscriptions are really required. Debt, £24,000. Secretary, Mr. Adrian Hope.

**Victoria Hospital for Children**, Queen's Road, Chelsea, S.W. (and Victoria Convalescent Home, Broadstairs).—The committee are appealing for special donations to the fund for the erection of a new building at a cost of £25,000. The hospital further seeks subscriptions to help it to carry on its work. Secretary, Mr. A. Cameron Skinner.

### WOMEN.

**Chelsea Hospital for Women**, Fulham Road, S.W.—This hospital (52 beds) is entirely without endowment or reserve funds, and greatly needs legacies and new annual subscriptions. There is a Convalescent Home (22 beds) at St. Leonards-on-Sea, but it is not restricted to those who have been patients in the hospital. Secretary, Mr. Herbert H. Jennings.

**Grosvenor Hospital for Women and Children**, Vincent Square, Westminster, S.W.—This hospital, hitherto inadequate and ill-contrived, has been recently reconstructed with modern improvements. Increased support is needed, two wards being still closed for want of funds. Secretary, Mr. H. F. Wilkinson.

**New Hospital for Women**, 144, Euston Road, N.W.—Only a want of funds prevents the committee from providing the beds necessary to treat the numerous cases waiting for admission to this hospital. Each bed costs about £75 a year, and an appeal is made to generous friends to come to the aid of the committee. Secretary, Miss M. M. Bagster.

**Royal Hospital for Children and Women**, Waterloo Bridge Road, S.E.—599 in-patients and 8,777 out-patients were treated during 1898. Secretary, Mr. Thos. S. Conisbee.

**Samaritan Free Hospital for Women and Children**, Marylebone Road, N.W.—Some £7,000 per annum is required to maintain this hospital, of which only £1,700 can be relied upon in annual subscriptions. The committee would, therefore, like to see a great addition to the list of annual and life subscriptions. Secretary, Mr. W. G. King.

**The Hospital for Women**, Soho Square, W.—There are 61 beds in constant use, and, as the hospital possesses no endowment, funds for their maintenance are much needed. The committee very earnestly appeal for additional annual subscriptions. Secretary, Mr. David Cannon.

### MISCELLANEOUS.—SPECIAL.

**Central London Ophthalmic Hospital**, Gray's Inn Road, W.C.—This hospital, which has treated over 400,000 patients during the fifty-six years of its existence, is in much need of funds. Secretary, Mr. J. G. Bryant.

**Dental Hospital of London**, Leicester Square, W.C.—The present hospital, which has been enlarged to its utmost extent, is quite inadequate, both in accommodation and sanitation, for the large number of patients. A new hospital is, therefore, being built on a very suitable site, almost adjoining the present hospital, and funds, for which an appeal has for some time been before the public, are still urgently needed. Secretary, Mr. J. Francis Pink.

**London Lock Hospital and Rescue Home**, Harrow Road, W.—A very large expenditure has been incurred this year, falling little short of £4,000, in the reconstruction of the drainage, the improvement in nurses' accommodation, and other things which it was difficult for the board to avoid taking in hand. Whilst this necessary outlay has improved the hospital immensely, yet it has quite exhausted the funds, and this expenditure must be met at once. Secretary, Mr. A. W. Cruikshank.

**London Fever Hospital**, Liverpool Road, Islington, N.—The institution is dependent upon voluntary support, and donations and subscriptions will be gratefully received, especially as the alterations and additions to the hospital, now being carried out, and the building of a convalescent home, are taxing to the utmost the resources of the institution. Secretary, Major W. Christie.

**National Orthopædic Hospital**, 234, Great Portland Street, W.—The hospital's pressing needs are, first, the receipt of £500 in donations to prevent heavy deficit on year's accounts, and then to increase its list of annual subscribers to three times its present amount. Secretary, Mr. H. J. Tresidder.

**Royal London Ophthalmic Hospital**, City Road, E.C.—An urgent appeal is made by the board of management for funds in support of this institution. The patients numbered in 1898: In-patients, 2,227; out-patients, 25,731. All patients are admitted free, and without letters of recommendation. Secretary, Mr. Robert J. Bland.

**Royal Orthopædic Hospital**, 297, Oxford Street, W.—During the period of more than 60 years since this hospital was founded more than 81,000 patients have benefited, many of whom entered helpless cripples and left in a condition to earn their own livelihood and support those dependent upon them. The committee require the sum of £2,000 before the close of the year. Every bed is now occupied. Secretary, Mr. Tate S. Mansford.

**St. Luke's Hospital**, Old Street, London, E.C.—This well-known institution has been treating mental diseases for the past 150 years. Is now solely devoted to our great

"middle-class" population whose means are limited. Two hundred beds are nearly always in use, and over 25,000 patients have been treated since the opening. The convalescent home in connection with the hospital is at St. Lawrence-on-Sea. Extensive improvements to increase the comfort of the patients have this year been completed, hence funds are urgently needed to maintain the charity, which is performing a special work to a most deserving section of our great community. Secretary, Mr. W. H. Baird.

### GENERAL CHARITIES.

**Association for the Oral Instruction of the Deaf and Dumb.**—The first association to publicly introduce into the United Kingdom the German or pure oral system for teaching deaf and so-called dumb children to speak *viva voce*, and to understand the spoken words of others by lip-reading. The expenses, which are very heavy, are met by voluntary contributions and fees, which fall short of what is needed by about £700 per annum. Offices, 11, Fitzroy Square, W.

**Bethnal Green Free Library, E.**—The poverty in the surrounding neighbourhood compels the committee to make an urgent appeal for funds to meet outstanding liabilities. Contributions will be thankfully received by the Librarian, at the Bethnal Green Free Library, E ; or by the Treasurer, Mr. F. A. Bevan, 54, Lombard Street, E C.

**British Home for Incurables,** Crown Lane, Streatham Common, S.W. Instituted in 1861.—This home is intended for those who, having once been in a position of comfort, are now in necessitous circumstances, and are either bedridden or greatly dependent on others in the various offices of life. Secretary, Mr. R. G. Salmond, 72, Cheapside, E.C.

**City of London Truss Society,** 35, Finsbury Square, E.C.—At the present time about 10,000 of both sexes and all ages are treated annually. The premises have recently been enlarged to provide for the increased number of patients, and additional funds are greatly needed to meet the growth in the expenditure. Secretary, Mr. John Whittington.

**Irish Distressed Ladies' Fund,** 17, North Audley Street, W.—Relief is given independently of any question of politics or religion ; employment is found, when possible, for those able to work ; pecuniary help is given tc the aged and infirm ; and the education of children is paid for.

**London Orphan Asylum,** Watford.—Established in 1813, the institution has maintained, clothed, and educated 6,150 orphan and fatherless children from every part of the United Kingdom, being the children of professional men, of principals engaged in manufactures, commerce, or trade, or of mercantile or other clerks and others of like class in life. These children have been given a sound commercial education, enabling them to fill positions in life with credit to themselves and their old school. Five hundred such boys and girls are now in the institution, one house, capable of accommodating 50 children, being unoccupied for want of funds. Nearly £14,000 has to be obtained each year from voluntary sources. New annual subscriptions and donations will be gladly received. Secretary, Mr. H. C. Armiger, 21, Great St. Helen's, E.C.

**London Schools Dinner Association.**—In view of the attempts which are made to throw upon the rates burdens which charity is competent to bear, attention is especially directed to the work of this association. It makes grants to provide cheap or free meals for necessitous children attending the public elementary schools of London, and is doing an excellent work. The annual income now averages about £1,500. Further subscriptions will be gratefully received to enable the association to be in a position to make immediate grants in cases of proved necessity (recommended by responsible local committees. Secretary, Mr. T. A. Spalding, 37, Norfolk Street, Strand, W.C.

**London Society for Teaching the Blind,** 10, Upper Avenue Road, Hampstead Road, N.W.—This society educates children, blind and partially blind, in general and technical instruction, viz., music, piano tuning, basketmaking, needlework, &c. Both sexes and all denominations and from all parts of the country received. The cost of carrying out this work is £2,800 a year, and the income of this society falls short of this amount by £600. Secretary, Captain G. G. Webber, R.N.

**Mary Wardell Convalescent Home for Scarlet Fever,** Stanmore, Middlesex.—This being the only home for convalescents from scarlet fever, the demand on its 40 beds is great. The committee have been compelled by the District Council to reconstruct the drainage system of the home in order to connect it with the public sewer which has recently been laid down, and funds are urgently wanted to meet this enforced expense. A demand for the first instalment of £200 has been received, but there is no money to meet it. Subscriptions to Miss Mary Wardell.

**Orphan Working School,** Haverstock Hill, N.W., and Hornsey Rise, N. Offices, 73, Cheapside, E.C.—A national and undenominational institution which maintains 500 children, varying in age from infancy to fourteen or (in special cases) fifteen years. It is greatly in want of funds at the present time. Secretary, Mr. Algernon C. P. Coote, M.A.

**Royal Albert Orphan Asylum,** Bagshot, Surrey.—The work of this institution, which had been curtailed owing to a falling-off in contributions, has again increased, and the maximum amount of relief is now being afforded. Special attention has been devoted to the reduction of a mortgage of £10,950, and a small amount (£300) has been repaid. It is hoped that in the continued efforts of the management to further reduce the heavy balance with which it is saddled generous assistance will be forthcoming. Secretary and superintendent, Mr. H. W. Tatum, King William Street, E.C.

**Royal Association in Aid of the Deaf and Dumb,** Oxford Street, W.—The objects of this society are to visit deaf and dumb persons at their own homes, and to assist them in sickness and distress. During the past year 3,559 visits were paid to the deaf and dumb, 849 visits were made to employers and others on their behalf, 205 were relieved, and 67 provided with work and apprenticed. An increased reliable income is needed to maintain and extend the present work. Secretary, Mr. Thomas Cole.

**Royal Blind Pension Society,** 237, Southwark Bridge Road, S.E.—This society provides pensions, by monthly instalments, for 1,030 blind people. Further contributions, particularly annual subscriptions, are required to enable it to give aid to 200 candidates who are approved as eligible for charitable assistance. Secretary, Mr. W. E. Terry.

**Royal Sea Bathing Hospital** (founded at Margate, 1791).—This hospital was founded for the relief of tubercular and other diseases requiring sea air and sea bathing. The system of open-air treatment has been carried out for many years past whenever suitable. The hospital is open all the year, and the full number of beds (150) is now available, and the average number occupied daily during the year is 125. Secretary, Mr. A. Peirce, 30, Charing Cross, S.W.

**Royal Alfred Aged Merchant Seamen's Institution** (established 1867) is the only institution in the United Kingdom which gives, irrespective of rank, creed, ports of service, or place of abode, a home or a pension to the British merchant sailor when old and destitute. Annual subscriptions do not reach £4,000 per annum, and the annual disbursements (including out-pensions and total support of inmates) are £7,000. Secretary, Mr. J. Bailey Walker; office, 58, Fenchurch Street, E.C.

**Shipwrecked Fishermen and Mariners' Royal Benevolent Society,** 26, Suffolk Street, Pall Mall East, S.W.—The work done by this society includes the granting of relief to shipwrecked persons, to widows and orphans of members and others, to destitute seamen, &c.; grants towards meeting loss of clothes or boats, assistance in old age or in case of infirmity. Over £24,290 was expended in relief in 1898 to relieve 11,010 persons. Income £22,783. Secretary, Mr. Gerald E. Maude.

